# Phoenix from the Ashes: Fire, Torpor, and the Evolution of Mammalian Endothermy

**DOI:** 10.3389/fphys.2017.00842

**Published:** 2017-11-02

**Authors:** Fritz Geiser, Clare Stawski, Chris B. Wacker, Julia Nowack

**Affiliations:** ^1^Centre for Behavioural and Physiological Ecology, Zoology, University of New England, Armidale, NSW, Australia; ^2^Department of Integrative Biology and Evolution, Research Institute of Wildlife Ecology, University of Veterinary Medicine Vienna, Vienna, Austria

**Keywords:** torpor, evolution of endothermy, K-Pg boundary, fire, development of thermoregulation

## Introduction

The evolution of endothermy in mammals and birds has been widely debated. Endothermy is characterized by high endogenous heat production via combustion of metabolic fuels. This differs from ectothermy in most living organisms, which generally do not produce substantial amounts of internal heat for thermoregulation (Tattersall et al., 2012; Withers et al., 2016). Endogenous heat production is energetically very costly. In comparison to ectothermic terrestrial vertebrates, namely the amphibians and reptiles, the minimum metabolic rate (MR) of normothermic or homeothermic (high constant body temperature, T_b_) animals at rest is about 4–8-fold higher in the endotherms. This difference is even more pronounced at low ambient temperatures (T_a_) at which the T_b_ of ectotherms follows T_a_, and the MR decreases to even lower levels. In contrast, the T_b_ of homeothermic endotherms remains high and constant over a wide range of T_a_. Therefore, to compensate for increased heat loss at low T_a_, MR of especially small mammals and birds must increase substantially and can be 100-fold or more of that in ectotherms (Bartholomew, 1982). Of course this high MR requires a substantial uptake of food and in endotherms much of this chemical energy is simply converted into heat for thermoregulation rather than growth or reproduction as in ectotherms.

Endothermy, however, brings a number of advantages. Most often discussed are higher stamina and peak performance of muscle due to a better oxygen and fuel delivery system (Bennett and Ruben, 1979; Nespolo et al., 2017), fast assimilation and growth rates due to better processing of food and improved metabolic machinery or production of larger litters via increased parental care (Koteja, 2000; Farmer, 2003). While all of these hypotheses have merit, they also have some limitations. The argument that the “aim” of endothermy was primarily not for maintenance of high T_b_, but rather an increase in aerobic performance, seems a chicken and egg question to some extent, because obviously improvements of thermogenesis and aerobic performance go hand in hand, and both would have increased together over time (Farmer, 2003). Similarly, the hypothesis regarding the function of endothermy in facilitating improved reproductive output via increased metabolism and homeothermy, although correct for many species, also has several potential shortcomings with regard to its generality. A number of mammalian species have been observed that show little or no increase in metabolism during reproduction as, for example, a monotreme and some marsupials (Nagy and Suckling, 1985; Holloway and Geiser, 2000; Nicol, 2017). To some extent this is because in endothermic monotremes and marsupials, conception to weaning times are about twice of those in placental mammals (Lee and Cockburn, 1985) and therefore energetic demands of reproduction are extended over prolonged periods. In contrast, in gravid ectothermic lizards MR increases significantly (Angilletta and Sears, 2000), and reproductive tegu lizards can maintain an increased T_b_-T_a_ differential via increased heat production (Tattersall et al., 2016). Therefore, although the argument makes sense and is correct for some endotherms with very high reproductive outputs and therefore energy and nutrient requirements, the relative cost for reproduction does not always differ between ectotherms and endotherms. Further, more and more species from all mammalian subclasses and also some birds even during reproduction are “heterothermic endotherms” (Greek *hetero* = other or different; *therme* = heat). Heterothermy in endotherms can be seen as a large temporal fluctuation of T_b_ above and below the homeothermic mean in large mammals (Hetem et al., 2016). However, in the context of our review heterothermy refers to torpor, which is a reduction of T_b_ by >5°C below the resting T_b_ (Ruf and Geiser, 2015) and/or a reduction of the metabolic rate (MR) below the basal MR (Geiser and Ruf, 1995), occurring predominantly in small endotherms. Importantly, even during the state of torpor these mammals and birds do not abandon endothermy as they can defend their T_b_ against a large T_b_-T_a_ differential and can rewarm from torpor using endogenously produced heat (Ruf and Geiser, 2015). These heterothermic endotherms may express torpor during pregnancy and/or lactation and even then show pronounced reductions of MR and T_b_ (McAllan and Geiser, 2014; Stawski and Rojas, 2016). When torpor is used in pregnant mammals, the period of pregnancy is usually extended by the time the animal spends in torpor, which can be by days or even weeks (Racey, 1973; Willis et al., 2006). Nevertheless, torpor expression during reproduction can have positive aspects. For example it can enable reproduction on limited resources, permits survival of adverse conditions during reproduction and can delay parturition until thermal conditions are more benign for mother and offspring (Geiser and Masters, 1994; Willis et al., 2006; Morrow and Nicol, 2009; Stawski, 2010). Clearly, some endotherms are willing to trade a fast development and growth rate for an increased chance for survival of offspring and mother, thereby also increasing their fitness.

Interestingly, the assumption of most discussions about the evolution of endothermy seems to be that reptilian ectothermy evolved into mammalian and avian endothermy by some stepwise increase in metabolism permitting an intermediate homeothermic T_b_ of around 20–30°C that went hand in hand with the growth in size and/or fur (Crompton et al., 1978; Ruben, 1995). The problem with this interpretation is, however, that homeothermy with a low MR even at low T_b_ is highly unlikely under most thermal conditions including the slightly warmer conditions in the Cretaceous (Royer et al., 2004) in small endotherms like the ancestral mammals for a number of reasons: (1) Even when the earth was warmer, it still will have experienced daily and yearly fluctuations in T_a_; now tropical areas can get rather cold in winter and many animals use torpor under these conditions (McKechnie and Mzilikazi, 2011; Dausmann, 2014). (2) A slight increase in MR, no matter what its purpose was, is insufficient for maintenance of a constant high or even slightly elevated T_b_ when the T_b_-T_a_ differential is large as for example at night (many small mammals can maintain a T_b_-T_a_ differential of >40°C requiring a manyfold increase in MR). (3) The lower critical temperature of the thermo-neutral zone below which MR must increase substantially if T_b_ is to be maintained in most small mammals is ~30°C or more, i.e., even when it is warm they must defend T_b_ against a substantial loss of heat (Riek and Geiser, 2013). (4) An intermediate T_b_ would have in fact interfered with high maximum heat production (Currie et al., 2015) and hindered a contribution of MR to thermoregulation. (5) Extant terrestrial ectotherms show large daily fluctuations in T_b_ and it is highly probable that the partially endothermic ancestors did exactly the same.

With regard to the asteroid strike causing the K-Pg extinctions 65 mya, a severe challenge for endotherms, the cost for thermoregulation in a small terrestrial homeothermic mammal would have been too high to survive the post-asteroid fires and the ensuing impact winter *in situ*. Consequently, the few species that did survive must have had physiological as well as behavioral adaptations to do so.

In this paper we discuss how, from a functional point of view, endothermy could have been attained. We use data both from extant developing as well as adult mammals to support our arguments. Data for developing animals are especially useful in the context of examining the transition from ectothermy to endothermy because altricial mammals and birds, the vast majority of all species, actually do show how an increase in metabolism from a largely ectothermic to an entirely endothermic organism is functionally and morphologically possible (Dawson and Evans, 1960). Thus, our arguments are founded on biologically relevant facts from thermal energetics data unlike many others that had to be largely speculative because the basic physiological mechanisms for thermoregulation like shivering and non-shivering thermogenesis are not preserved in the fossil record, although evidence for insulation can be (Withers et al., 2016). Developmental data may not reveal exactly the same pathways and sequences as those during evolution, but rather show biologically likely and possible scenarios. This is a far more promising approach than relying exclusively data on adults that do not show the transient states seen during development of altricial endotherms. Finally, we discuss how mammals could have managed to survive the post-impact winter following the asteroid strike. New data on post-fire behavior and physiology of terrestrial mammals provide examples for possible survival avenues on that topic. With regard to the latter we mainly discuss mammals in our paper because birds can fly and thus avoid or select areas that were more or less affected by the asteroid strike.

## Were ancestral endotherms homeothermic or heterothermic?

Imagine a reptile with a MR that was increased by 10% above that of its ancestor. How would this have affected its biology? It may have allowed some increased stamina to obtain food because an improved delivery system for oxygen and fuels would have enhanced both locomotion and the capacity for shivering. However, the most obvious advantage would have been an increase in the time the animal was able to be active with an elevated T_b_ when T_a_ was falling in the evening and perhaps at the beginning of warm nights. This temporal pattern has been observed in several small extant nocturnal mammals that show extremely brief activity and foraging periods of an hour or two early in the evening when they maintain a high T_b_ (Warnecke et al., 2008; Körtner and Geiser, 2009; Stawski and Geiser, 2010). Later at night, as for its fully ectothermic ancestors and extant reptiles, the mammalian ancestor, as do its extant relatives, would have become cold, because a small increase in metabolism would not have been high enough for a sustained elevated T_b_ throughout the night. Although the rather large (~2,000 g) reproductive tegu lizards could maintain a raised T_b_ by using increased heat production and peripheral vasoconstriction (Tattersall et al., 2016), T_b_ still fluctuated during the day and the average T_b_-T_a_ differential of 6–7°C was well below that of the often >30°C T_b_-T_a_ differential observed in modern normothermic mammals and birds even at body mass of <5 g.

On the next morning, the metabolism of the ancestral endotherm would not have been high enough to raise T_b_ endogenously because that requires a manyfold increase of MR, or high metabolic scope, which would have been further hindered by the low T_b_ (Currie et al., 2015). However, it could rewarm in the sun or with an increasing T_a_, as do many extant torpid mammals with a very low MR that remains below BMR (i.e., near the values of reptiles) for much of the rewarming process (Lovegrove et al., 1999; Geiser et al., 2002, 2004; Dausmann et al., 2004). With time an even higher metabolism would have been selected in ancestral endotherms via increased shivering and non-shivering thermogenesis (Oelkrug et al., 2015) together with an external cover of fur or feathers resulting in a prolongation of periods with a high and constant T_b_. Importantly, and in contrast to what was believed in the past (Cowles, 1958), fur cover in living animals does not necessarily make basking energetically ineffective (Geiser et al., 2004), because unlike dead fur coats used to cloak ectothermic lizards (Cowles, 1958), mammals can adjust the angle of hair to maximize heat gain (Wacker et al., 2016). Basking mammals during normothermia can maintain resting MR near basal MR values over a wide range of T_a_ well below the thermo-neutral zone and can reduce rewarming cost by up to 75% in comparison to endothermic rewarming from torpor (Geiser and Drury, 2003). Moreover, and also in contrast to what was believed in the past (IUPS Thermal Commission, 2003), torpid animals can travel at low T_b_ of ~15°C or less to move to basking sites and their ability to do so also aids in predator avoidance (Rojas et al., 2012).

Fortunately, we do have examples of extant mammals supporting these evolutionary arguments on the use of heterothermy during the evolution of endothermy. Marsupial dunnarts (*Sminthopsis* spp.), small insectivorous mammals, are born naked at a small body mass/size with low endogenous heat production (Wacker et al., 2017). They do not need a high heat production because initially they are kept warm within their mother's pouch and in birds and placental mammals brooding and nests serve a similar purpose. At the time of pouch exit young dunnarts are partially furred, but like other small marsupials are still only partially endothermic as they cool rapidly when exposed to low T_a_ (Morrison and Petajan, 1962; Geiser et al., 2006; Wacker et al., 2017). Interestingly, almost competent endothermic thermoregulation develops soon after pouch exit and the young can maintain a high T_b_ for some of the night, but in the second part of the night they enter an apparent bout of torpor (Wacker et al., 2017). They do so despite the fact that they lack high enough endogenous heat production to rewarm from low T_b_. Instead these juveniles seem to “know” that they can rely on behavioral thermoregulation and bask under a radiant heat source to raise T_b_ to high level as their ectothermic ancestors must have done, and extant reptiles continue to do, on sunny days. So here we have a highly plausible and functionally possible model as to how endothermy could have evolved via a transient partially endothermic heterothermic phase requiring behavioral thermoregulation, but permitting some crepuscular and nocturnal activity and foraging to avoid dinosaur predators (McNab, 2002). The ability of using a combination of behavioral and physiological thermoregulation to reach high T_b_ would have maximized biological functions during a somewhat prolonged activity phase. This in turn would have increased stamina and, if nutrition was sufficient, would have allowed production and fast growth of many young.

Importantly, torpor during reproduction and development is not restricted to marsupials. Data on torpor during incubation, brooding, pregnancy, and lactation are now available for nightjars, hummingbirds, echidnas, several marsupials, tenrecs, hedgehogs, bats, carnivores, primates, and rodents (McAllan and Geiser, 2014; Stawski and Rojas, 2016). During development, although published work is scant, torpor has been observed in several birds, marsupials, rodents, and shrews and there is some evidence that it also occurs in bats (Geiser, 2008; Giroud et al., 2014). This use of torpor during reproduction and development in so many diverse vertebrates lends further support to our arguments.

The proposal that homeothermy in mammals must have evolved via heterothermy makes functional sense, because this avenue provides a plausible explanation as to how metabolism could have increased gradually over time (Lovegrove, 2017). While torpor patterns may have not been the same as those expressed in modern mammals with the ability to inhibit MR and defend T_b_ during torpor (Geiser, 2008) it still would have been advantageous during the transition to endothermy. Heterothermy would have permitted a low T_b_ and energy conservation during cold exposure, and passive rewarming from low T_b_ before the activity phase would have been possible with a relatively low MR (Geiser and Drury, 2003; Grigg et al., 2004). Consequently, prolonged activity and foraging during the first part of the night to avoid predation appears to be the initial selective advantage of an increased MR in ancestral endotherms. Over time, activity would have been extended and perhaps in some species homeothermy evolved whereas others continued to be heterothermic, which is consequential for our next chapter addressing mammalian survival of a calamity that caused the extinction of many terrestrial animals.

## The function of mammalian heterothermy at the K-Pg boundary

The asteroid impact at the Cretaceous-Palaeogene (K-Pg) boundary, about 65 million years ago, ended the era of dinosaurs, but was the beginning of the diversification of extant mammals. Geological evidence suggests that the asteroid caused global wildfires that killed all terrestrial life unable to seek safe refuge mainly underground (Morgan et al., 2013). The disappearance of the dinosaurs opened new niches and permitted a rapid radiation of mammalian lineages (O'Leary et al., 2013). However, before mammals could diversify they had to (i) survive the fires caused by the asteroid impacts and (ii) survive the post-impact winter that lasted for many months. As for the evolution of endothermy *per se*, heterothermy and torpor expression were likely crucial for both (Lovegrove et al., 2014; Nowack et al., 2016).

A homeothermic small mammal may have had the ability to survive the immediate effect of the fires if hidden underground, however, it would not have been able to survive without food for months during the post-impact winter. The only way for small sedentary endotherms to achieve this without enormous food caches is the ability to enter torpor, which would have permitted these mammals to stay inactive and hidden for long periods without the need to forage (Turbill et al., 2011). During multiday torpor in hibernators, the metabolic rate can be reduced to <5% of the basal MR and T_b_ often falls to near 0°C, but substantial energy saving can also be achieved at relative high T_b_ (Tøien et al., 2011; Ruf and Geiser, 2015). Huddling in groups could have further enhanced energy savings (Arnold, 1993; Gilbert et al., 2010; Eto et al., 2014; Nowack and Geiser, 2016). Although it is widely accepted that hibernating mammals can survive without food for about 6 months, recent data have shown that some can do even better than that. For example, captive fattened eastern pygmy-possums (*Cercartetus nanus*) a small marsupial, can survive when hibernating at low T_a_ for up to an entire year on stored fat despite periodic arousals (Geiser, 2007). Similarly, dormice (*Glis glis*) during non-reproductive years can hibernate for up to 11 months per year in the wild and in captivity (Bieber and Ruf, 2009; Hoelzl et al., 2015). Tenrecs (*Tenrec ecaudatus*) and lemurs (*Cheirogaleus* spp.) can hibernate for prolonged periods even in the tropics (Dausmann et al., 2004; Blanco et al., 2013; Lovegrove et al., 2014). Such periods would have been sufficient for some individuals to survive the post-impact winter.

**Figure 1:**
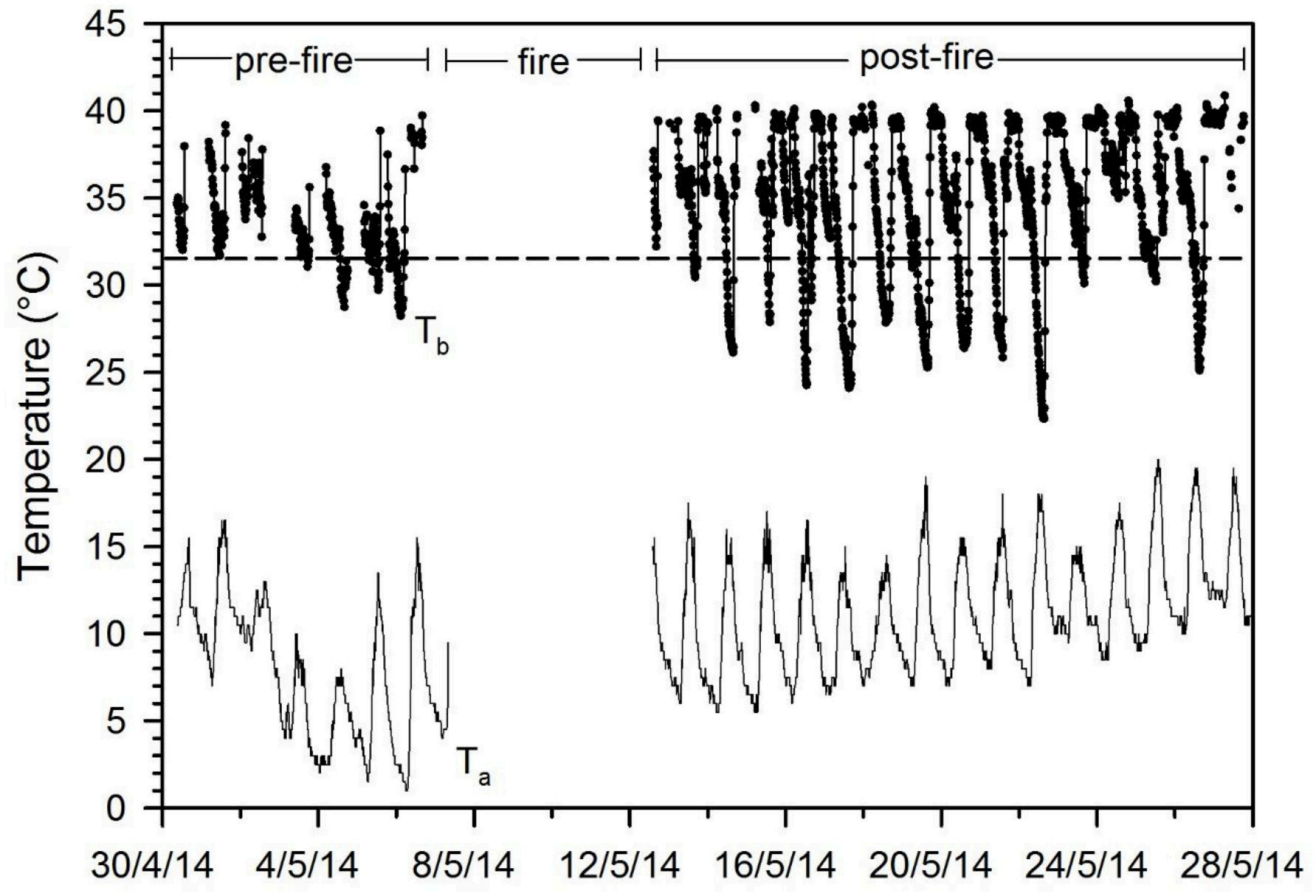
Body temperature (T_b_) and ambient temperature (T_a_) fluctuations in the free-ranging marsupial *Antechinus stuartii* in the late Austral fall before and after a controlled forest fire. Access during the fire for data collection was not permitted. Note the substantial increase of the T_b_ fluctuations and prolonged duration of torpor bouts after the fire although T_a_ increased somewhat during that time in comparison to before the fire (data from Stawski et al., 2015). The horizontal dashed line is the torpor threshold.

New evidence also suggests that torpor is used extensively to deal with fires or the scorched post-fire environment in extant terrestrial mammals. Echidnas, *Tachyglossus aculeatus*, egg-laying mammals that have many ancestral functional and morphological traits (Nicol, 2017), hide and enter torpor during forest fires (Nowack et al., 2016). Before the fire echidnas expressed brief and shallow bouts of torpor whereas after the fire animals entered prolonged periods of torpor although T_a_ was rather mild. Interestingly animals also reduced activity but remained within their original home range. Similarly, antechinus (*Antechinus stuartii* and *A. flavipes*), small insectivorous marsupials, increased torpor expression and duration, both after a controlled forest fire for fuel reduction (Figure 1) as well as a wildfire, and at the same time decreased daily activity (Stawski et al., 2015; Matthews et al., 2017). The reduction in activity was mainly achieved by reducing daytime activity, likely to avoid exposure to predators in a habitat with little vegetation cover, as would have been the case during the post-impact winter caused by the asteroid. The post-fire increase in torpor use in extant mammals was initially assumed to be to a large extent related to a decrease in food availability that typically follows a fire. However, recent data show that the presence of charcoal-ash substrate and smoke enhances mammalian torpor use beyond that simply induced by food restriction, suggesting that these post-fire cues signal a period of imminent food shortage to the animals (Stawski et al., 2017). This is further evidence that during the post-impact winter, when mammals would have been exposed to food shortage, a habitat with limited cover, ash/charcoal substrate and perhaps smoke, torpor expression would have increased to minimize energy expenditure and foraging requirements, providing an avenue for survival to mammals. Birds also suffered extinctions during the K-Pg calamity, but the survivors likely relied on mobility rather than prolonged torpor because only one extant avian species is known to hibernate (Brigham, 1992) in contrast to the many mammalian hibernators (Ruf and Geiser, 2015). However, it cannot be excluded that birds also employed torpor to some extent when faced with energetic and thermal challenges as is known for many diverse extant species (Brigham, 1992; McKechnie and Lovegrove, 2002).

More and more evidence is accumulating on the adaptive advantages of heterotherms over homeothermic species. Heterothermic species do not only use torpor to survive seasonal energetic and thermal challenges, but can also endure the consequences of unpredictable energy bottlenecks or natural disasters (Nowack et al., 2015, 2016, 2017) and consequently have a lower risk of becoming extinct (Geiser and Turbill, 2009; Turbill et al., 2011; Hanna and Cardillo, 2014). Ancestral mammals were small and nocturnal and presumably had a relaxed endothermic thermoregulation, expressing torpor during the colder periods of the day and possibly also were able to use multiday hibernation for highly effective energy conservation (Grigg et al., 2004). Many of today's heterotherms hibernate in underground burrows (Arnold et al., 1991) that would allow survival largely independent of the conditions on the Earth's surface, as would have been the case during the K-Pg boundary. Thus both during the initial evolution of endothermy in birds and mammals and the survival of mammals during the K-Pg boundary, heterothermy likely played a key role because it permitted an intermediate metabolism during the evolution of endothermy as well as prolonged survival without food at the K-Pg boundary.

## Author contributions

All authors listed, have made substantial, direct and intellectual contribution to the work, and approved it for publication.

### Conflict of interest statement

The authors declare that the research was conducted in the absence of any commercial or financial relationships that could be construed as a potential conflict of interest.
